# Prioritizing qualitative meta-synthesis findings in a mixed methods systematic review study: A description of the method

**DOI:** 10.1017/rsm.2024.8

**Published:** 2025-04-01

**Authors:** Robin Coatsworth-Puspoky, Wendy Duggleby, Sherry Dahlke, Kathleen F. Hunter

**Affiliations:** 1 School of Nursing, York University, Toronto, ON, Canada; 2 Faculty of Nursing, University of Alberta, Edmonton, AB, Canada

**Keywords:** cross-study table, mixed methods systematic review procedure, prioritizing meta-synthesis findings, validity of integrated study findings, vote counting, qualitative evidence synthesis, synthesis of qualitative and quantitative evidence, matrix to compare synthesized evidence, integration of synthesized evidence

## Abstract

**Aim(s):**

To describe a sequential mixed methods review method that prioritized synthesized qualitative evidence from primary studies to explain the complexities of older persons with multiple chronic conditions’ unplanned readmission experiences.

**Background:**

Segregated mixed methods review studies frequently prioritize quantitative evidence synthesis to examine the effectiveness of interventions; utilizing qualitative evidence to explain quantitative data. There is a lack of guidance about how to prioritize qualitative evidence.

**Results:**

Five procedural steps were developed to prioritize qualitative evidence synthesis. In Step 1, research questions were developed. In Step 2, databases were searched, studies were mapped to their method (qualitative or quantitative) and appraised. In Step 3, meta-synthesis and applied thematic analysis were used to synthesize extracted qualitative evidence about the psychosocial processes and factors that influenced unplanned readmission. In Step 4, quantitative evidence was synthesized using vote counting to determine the factors influencing unplanned readmission. In Step 5, a matrix was used to compare, determine the agreement between the qualitative and quantitative evidence, juxtapose findings, and uphold validity. Factors were mapped to the model of psychosocial processes and analytic themes.

**Conclusion:**

Prioritizing qualitative evidence synthesis in a mixed methods review study prioritizes participants’ experiences, perspectives, and voices to understand complex clinical problems from participants who experienced the event. Synthesizing and integrating evidence facilitates the construction of holistic new understandings about phenomenon and expands mixed methods systematic review methods.

**Implications:**

Prioritizing patients’ perspectives is useful for developing new client-centered interventions, establishing best practices for future reviews, generating theories, and expanding research methods.

## Highlights

### What is already known?

- Harden and Thomas developed a unique mixed methods systematic review (MMSR) design which prioritized the synthesis of quantitative evidence before the synthesis of qualitative evidence and integration of synthesized evidence. They proposed that the steps of their sequential MMSR could be adapted to prioritize synthesized qualitative evidence, but did not provide clear explanations, guidance, or examples.

### What is new?

- Documenting and illustrating the method and framework developed to prioritize qualitative evidence in a MMSR study addresses a gap in the literature, provides clarity about the procedure, provides a foundation to guide future reviews, and expands current understandings of MMSR designs and methodologies.

### Potential impact for *Research Synthesis* readers?

- Synthesized qualitative data from using a meta-synthesis and applied thematic analysis resulted in the construction of themes to describe psychosocial processes and qualitative factors, advancing the utilization and development of qualitative evidence synthesis (QES) within MMSR studies.
- Prioritizing synthesized qualitative evidence emphasizes and upholds the voices, language and subjective experiences of those (older persons) who experience the complex clinical problems, assisting researchers to construct a holistic understanding of the problem. Utilizing language that is consistent with older persons assists clinicians to develop a working relationship and interventions with patients to address patients’ health and social care needs.

## 1 Introduction

In health care, mixed methods systematic review (MMSR) studies are critical to the advancement of knowledge. Integrating synthesized qualitative and quantitative evidence using an MMSR study design helps researchers identify and explain findings that are consistent, inconsistent or contradictory, and the need for research to address complex clinical practices or health care issues and support the development of new research questions.^1^ ^–^ ^4^ Names of MMSR studies vary and include mixed research synthesis,^4^ mixed methods synthesis^5^ studies, mixed methods review,^6^ or systematic mixed study review.^7^ Although the names vary, researchers agree that the purpose of MMSR studies is to develop comprehensive understandings about complex health care issues comprised of clinical practices, interventions, and policies.^2^ ^–^ ^5^ ^,^ ^8^ ^–^ ^10^

MMSR studies are their own “distinct” research methodology “category”^5^ (p. 2) with established and systematic methods and procedures to design, review, synthesize, and integrate primary (qualitative, quantitative, and mixed methods) studies related to the same health care issue.^2^ ^,^ ^4^ ^,^ ^5^ ^,^ ^9^ Researchers using a MMSR method, integrate synthesized findings from qualitative and quantitative studies to construct a holistic or more complete response to a research question. For example, by integrating findings about the effectiveness of an intervention with qualitative evidence about the experience or meaning of the intervention, researchers can construct a holistic understanding about the intervention or practice.^3^ ^,^ ^5^ ^,^ ^11^ ^,^ ^12^

Some MMSR studies prioritize quantitative over qualitative evidence and others qualitative over quantitative evidence. The decision of whether or not to prioritize (synthesize first) findings from quantitative studies or qualitative studies in designing the study, is influenced by the review question.^13^ It may also reflect the preference of the reviewers or guidance that one method is not selected over another method and treated as separate entities.^4^ ^,^ ^14^ For example, to understand what was known about older persons with multiple chronic conditions’ (MCCs) experiences with unplanned readmission, Coatsworth-Puspoky et al.^15^ created an MMSR method that prioritized synthesized qualitative evidence because the overarching aim of the study was to understand older persons with MCCs’ unplanned readmission experiences within 30 days of discharge from the hospital.^15^ Harden and Thomas^5^ identified the idea of prioritizing synthesized qualitative evidence as an opportunity to “inform the boundaries of analysis,” “determine the variables for a subgroup analysis,” and expand the scope and knowledge about MMSR methods but did not outline a procedure, and only specified the analysis methods to synthesize quantitative data^5^(p. 31). Further, limited explanations and method exemplars of MMSR studies which prioritized synthesized qualitative research findings (qualitative evidence synthesis; QES)^4^ ^,^ ^16^ were found. As a result, the researchers used Harden and Thomas’^5^ sequential mixed methods synthesis method and framework as a template for the construction of a new MMSR method for the development of nursing knowledge.

## 2 Aims

This paper presents a new MMSR method and framework which expands the work of Harden and Thomas^5^ and addresses a gap in the literature about utilizing, developing, and prioritizing QES in MMSR studies.^16^ ^–^ ^18^ Following a critique of MMSR study designs and their key elements and Harden and Thomas’^5^ sequential MMSR, the method and procedural steps developed by Coatsworth-Puspoky et al.^15^ will be presented.

## 3 Background

MMSR research or synthesis studies mix, integrate, and synthesize primary research findings to answer and address complex health care issues and questions.^5^ Selecting the MMSR study design is influenced by the research question^5^ ^,^ ^12^ or concepts^14^ of the study design,^4^ and underlying assumptions and philosophy about the evidence.^4^ The concept or dimension central to MMSR studies includes the appropriateness of the analysis method to address the health care issue.^12^ The analysis method includes synthesis methods and sequence, data transformation (if necessary and when it will occur), and integration methods^2^ ^,^ ^4^ ^,^ ^14^ (p. 2111) in all design typologies.

Traditional MMSR study design typologies include segregated, integrated, and contingent.^2^ ^,^ ^4^ In segregated designs, qualitative and quantitative primary findings help researchers answer complementary research questions about the same phenomenon for the purposes of explaining and understanding.^4^ ^,^ ^5^ The way of synthesizing findings to address the research question is specific to the research approach or method (qualitative or quantitative). Qualitative and quantitative findings are synthesized separately. Once synthesis is completed for qualitative and quantitative findings, synthesized qualitative and quantitative findings are integrated. Before integrating synthesized findings, researchers must ensure findings are “related to each other” and “in the same domain of research,” or address “the same aspects of the domain”^4^ (p. 7). Determining how the study findings are similar or related influences researchers’ decisions about whether findings can be aggregated or configured or segregated.^4^ ^,^ ^19^ For example, configuration is indicated when researchers use findings that are dissimilar to make connections, relationships and frameworks. Further, the segregated design is indicated when the goal is to configure findings from qualitative and quantitative research into a “coherent whole” of an argument, relationships, or timeline of events^4^ (p. 7).

In integrated MMSR designs, findings from qualitative and quantitative data help researchers verify, expand, or discredit a topic. In contrast to the segregated designs, findings from one method (e.g., qualitative) are transformed into the other method (e.g., quantitative) and are not synthesized separately. Before the data are transformed, the researcher determines whether quantitative data will be “qualitized” or transformed into themes or whether qualitative data will be “quantified” or transformed into numbers.^14^ Integration occurs after the findings are transformed. Contingent MMSR designs are differentiated from both segregated and integrated designs as synthesized findings specific to each research method are part of a cycle that is used to answer questions and identify questions to pose for the next group of studies.^4^ With the aim of developing a typology of synthesis processes (methods and designs), Hong et al.^7^ identified two additional MMSR designs: convergent and sequential. Convergent MMSR designs are similar to Sandelowski et al.’s^4^ integrated or segregated MMSR review studies. In contrast, sequential synthesis MMSR studies are similar to Sandelowski et al.’s^4^ contingent MMSR design. In both of these designs, researchers sequence their analysis and use findings from one synthesis in one phase to inform the other findings and subsequent phases.^3^ ^,^ ^7^ ^,^ ^14^

The terms synthesizing and integrating are often interchanged but are two distinct methods of analysis used in MMSR studies.^4^ ^,^ ^20^ Synthesis is a process researchers use to bring qualitative, quantitative, and mixed methods findings together to address the same research question.^4^ Synthesizing is a method used first to analyze primary research findings and is common across MMSR study designs.^12^ The type of data and research question influence the researchers’ choice of synthesis method.^12^ ^,^ ^21^ Experts advise that the synthesis method be suitable to the research method (qualitative, quantitative) and implemented in a series of steps.^21^ Tricco et al.^22^ reviewed the literature (*n* = 409) and discovered 25 methods of data synthesis. However, less than 20% of researchers described how they operationalized the synthesis method they used (p. 22). In a review of the literature, researchers uncovered three elements that were lacking in the synthesis of data in MMSRs. These elements included details about the synthesis method used, how the synthesis was implemented, and documentation about the steps of the synthesis.^14^ Stern et al.’s^14^ findings, supportive of Noyes et al.’s^23^ conclusions, acknowledged that synthesis methods lacked development,^11^ testing, and evaluation.

To date, researchers have used MMSR studies to examine the effectiveness of clinical interventions across studies using synthesized qualitative evidence to support the synthesized quantitative evidence.^3^ The primary use of QES was to enhance the breadth of understanding people’s experiences or their medical conditions or interpret the reasons or factors that contributed to the success of interventions^1^ ^,^ ^2^ ^,^ ^14^ and to explain the “similarities and differences in language, concepts, images and other ideas around a target experience.”^24^ Both Harden and Thomas^5^ and Sandelowski et al.^4^ hypothesized that the QES could be prioritized to help researchers understand complex phenomenon, events, or processes, but offered little guidance about how to do this. In addition, exemplar MMSR studies where researchers prioritized and integrated synthesized evidence about people’s experiences or perspectives were not found in the literature as reviews do not specify which synthesis was completed first in their review title, abstract, or in the article. Further, using QES to address a research question to understand the complexity of the process was not found in the published literature. Harden et al.^11^ affirmed the scarcity of MMSR studies that integrate “qualitative and process evaluation evidence alongside quantitative evidence” and confirmed that most MMSR studies were related to intervention effectiveness (p. 71). To answer the research question about what the psychosocial processes of older persons with MCCs’ unplanned readmission experiences were like, synthesized qualitative evidence was used to identify the factors influencing older persons with MMCs’ unplanned readmission experiences. Harden and Thomas’ MMSR design was a template to develop a new MMSR design that prioritized QES.^15^

### 3.1 Harden and Thomas’^5^ **MMSR design**

Harden and Thomas’^5^ MMSR design is unique and they categorized it as “a sequential mixed methods design”^5^ (p. 31). Their MMSR design is differentiated from other systematic review designs by three features: first, construct a “range of questions” to understand what is known about the phenomenon; second, characterize the data and methods to synthesize qualitative and quantitative data as equal; and third, prioritize one evidence synthesis (qualitative or quantitative) over the other^5^ (p. 9). Prioritizing one evidence synthesis over the other influences how data are integrated, which evidence synthesis is interrogated by the other evidence synthesis, and in the final phase of analysis, how data are interpreted.

In Harden and Thomas’^5^ MMSR design, researchers used different methods to complete separate quantitative then qualitative syntheses before integrating the findings or evidence. The synthesis and integration of evidence occurred in three procedural steps. First, Harden and Thomas^5^ identified the review study purpose, primary research question, and two questions. The primary research question or overarching question addressed “what is known” about the phenomenon. Based on the researchers’ previous work, they hypothesized that analyzing the quantitative evidence would help them to differentiate the effectiveness of interventions and that non-intervention studies would help them understand the factors that contributed to people’s experiences with the interventions. The overarching research question determined which question was prioritized or “weighted” first and how evidence from each subquestion was integrated or used in evidence integration. Two questions were designed next to answer the overarching question and integrate synthesized qualitative and quantitative evidence. These questions ensured that separate synthesis occurred for qualitative and quantitative evidence and that synthesis methods were specific to each question. For example, when quantitative evidence was prioritized, qualitative evidence was used to inform the quantitative evidence. Integrating evidence from research questions occurs in two stages using a conceptual and methodological matrix.^5^

By prioritizing the quantitative evidence synthesis, researchers prioritized the “statistical power”^25^ of the intervention’s effectiveness, impact, and method of analyzing quantitative evidence.^14^ Using a meta-analysis assisted the researchers to examine the interventions and conclude that the interventions were effective.^5^ From the results of the meta-analysis, the researchers were only able to speculate about the findings and were not able to explain variations in the effect sizes in individual studies. If the QES were prioritized, the researchers’ foci would be on descriptions of experiences to determine themes and concepts that “tie a variety of experiences together, or that separate some participants from others”^25^ (p. 60). Key areas explored using QES include “context, process, and meaning” with the overall goal of understanding participants’ experiences related to “health and social experiences”^5^ (p. 6). The purpose of prioritizing the QES would be to inform the quantitative synthesis and identify variables.^5^

In the second step, Harden and Thomas^5^ selected a meta-analysis as the method to synthesize quantitative findings but argued that vote counting could also be used. In the second step, Harden and Thomas selected a meta-analysis as the method to synthesize quantitative findings but argued that vote counting could also be used. To answer the second research question, qualitative data were synthesized using thematic synthesis. “Line-by-line” coding from meta-ethnography was used to categorize the data (meaning and content), synthesize data by translating concepts across studies, and cluster codes based on their similarities and differences. Clustered codes were assigned a new code and meaning, creating descriptive themes. “Third-order interpretations” from meta-ethnography were constructed using the review questions about participants’ perspectives or experiences explaining the analytic themes. In this step, researchers developed themes related to participants’ perspectives and experiences and which they combined to develop interventions. Combined, analytic and descriptive themes facilitated the generation of “new interpretive constructs, explanations or hypotheses”^5^ (p. 1).

In the third step, two steps were completed to integrate quantitative findings with qualitative findings: integration and matrix. Integration functioned to “sum up” what is known about the phenomenon^4^ (p. 1). Harden and Thomas^5^ analyzed and evaluated whether trial interventions addressed or included the experiences or perspectives about the implications from their study findings. During the process of integration, quantitative data were compared and analyzed with the experiences of participants. The conceptual and methodological matrix used in the third synthesis step assisted researchers to formulate explanations and interrogate or test the finding from the first synthesis with findings from the second synthesis. A cross-study matrix was used to present a visual map and illustrate the synthesis and evaluation of matches (similarities), mismatches (dissimilarities), research gaps (not addressed), and intervention quality related to the overarching research question. The matrix assisted the researchers to compare the findings from each synthesis and to determine the agreement between the qualitative and quantitative evidence.^5^

Although Harden and Thomas^5^ argued that their sequential MMSR could be adapted to answer research questions that prioritize synthesized qualitative evidence, they did not provide clear explanations, guidance, and concrete examples to use when prioritizing QES. Further suggestions about how to prioritize synthesized qualitative evidence about the psychosocial processes experienced by older persons were not identified by Harden and Thomas^5^ or in MMSR literature. To address this gap in the methods literature, the procedural steps Coatsworth-Puspoky et al.^15^ used to synthesize qualitative and quantitative evidence and integrate synthesized evidence will be described and illustrated.^26^ Disseminating the steps of our procedure that prioritizes QES in an MMSR design expands our knowledge and facilitates understanding about using QES and integrated findings to influence social and health care practices.^27^

## 4 Prioritizing qualitative meta-synthesis findings in an MMSR study

**Figure 1:**
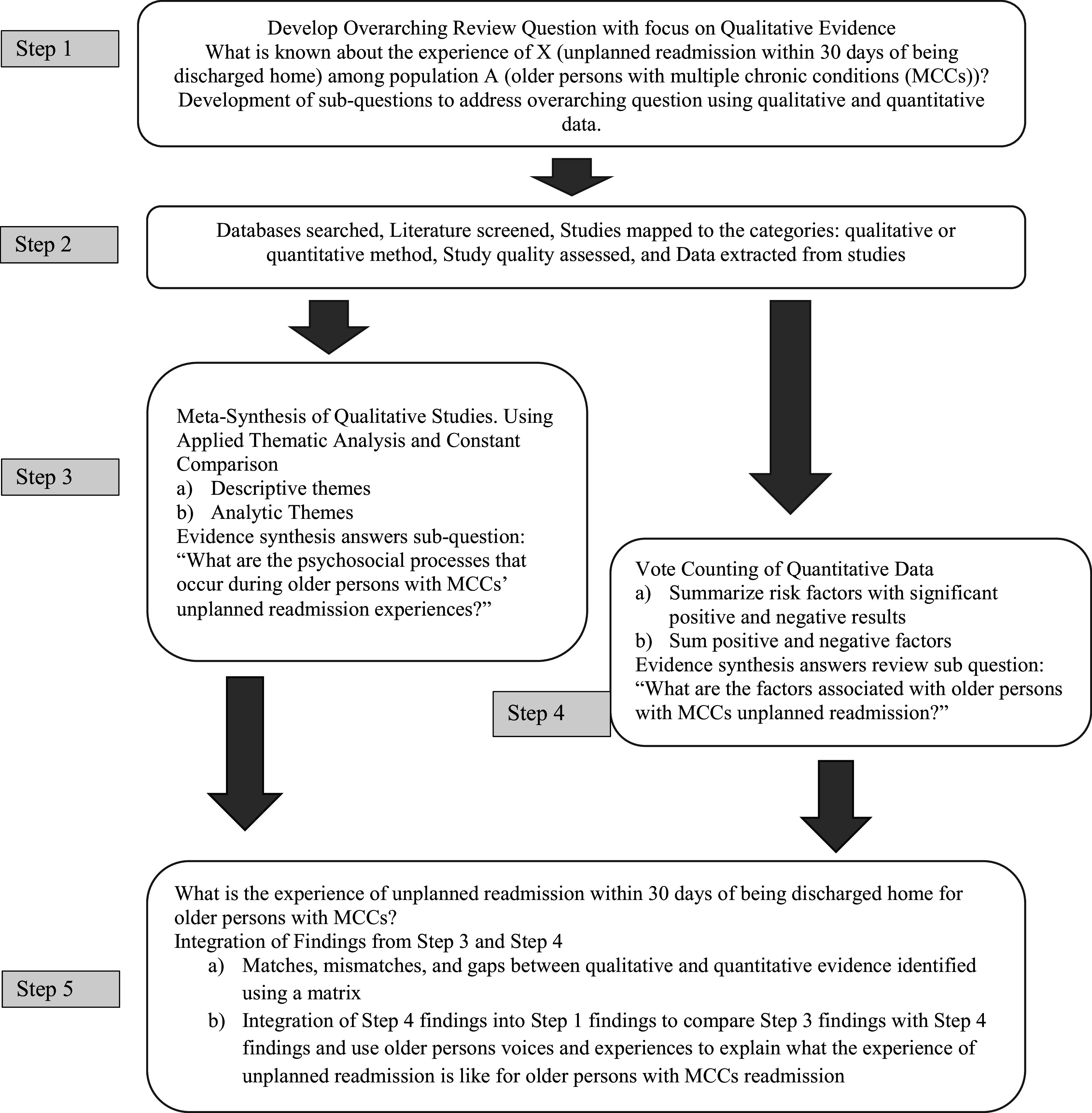
Prioritizing qualitative meta-synthesis findings in a mixed methods systematic review. Note: The review process outlined above is adapted from Harden and Thomas^5^ and Coatsworth-Puspoky et al.^15^

The procedure of prioritizing qualitative meta-synthesis findings progressed through five systematic steps. These steps included (1) formulation of the research questions; (2) systematic literature search, mapping of findings to their method (qualitative or quantitative), appraisal of selected primary studies, and extraction of data from selected studies; (3) synthesis of qualitative findings from primary qualitative and mixed methods studies using meta-synthesis and Guest et al.’s^28^ applied thematic analysis; (4) synthesis of quantitative findings from primary quantitative and mixed methods studies using vote counting; and (5) integration of synthesized qualitative and quantitative evidence using a matrix, juxtaposing findings, and mapping. Each step of the procedure used to prioritize meta-synthesis findings in an MMSR study is illustrated in Figure 1 and described in detail.

### 4.1 Step 1: Reserach question formulation

Before beginning the literature search, three research questions were formulated: an overarching question and two questions to guide analysis for Steps 3 and 4. The overarching research question determined the type of research that would be prioritized and influenced the method of analysis that would be used. For example, Coatsworth-Puspoky et al.^15^ sought to understand what the qualitative experience of unplanned readmission was like for older persons with MCC within 30 days of being discharged home. Understanding what the experience of unplanned readmission was like for older persons was the purpose of the study and was the overarching review question which was used to prioritize or sequence the synthesis of qualitative findings from primary qualitative and mixed methods research studies about older persons’ unplanned readmission experiences.

To address the overarching question, two separate questions were developed that upheld the “conventional binary distinction between qualitative and quantitative research”^5^ ^,^ ^4^ (p. 6). The first question, developed to prioritize QES (qualitative evidence from qualitative and mixed methods studies) and provide experiential evidence to answer the overarching research question, was What are the psychosocial processes that older persons with MCCs’ experience during unplanned readmission to hospital within 30 days of discharge home? To understand the psychosocial processes of and nuances influencing older persons with MCCs’ unplanned readmission experiences, the qualitative analysis was prioritized or sequenced first. The second question, developed to identify factors influencing the unplanned readmission experiences of older persons, was What are the factors that influence or are associated with older persons with MCCs’ unplanned readmission experiences? The factors were identified from quantitative evidence from quantitative and mixed methods studies.

### 4.2 Step 2: Systematic literature search, appraisal of selected primary studies, and data extraction

Inclusion and exclusion criteria were developed to search through the literature and ensure that the selected research studies addressed the same topic, older persons with MCCs’ experiences of unplanned readmission within 30 days of discharge^15^ (p. 5796). Two members of the research team (RCP and SD) used the inclusion and exclusion criteria and covidence to determine appropriate qualitative, quantitative, and mixed methods research studies to include from six databases for the first research question. The inclusion and exclusion criteria ensured that the included studies addressed the study focus of unplanned readmission experiences of older persons (over 60 years of age) with MCC within 30 days of discharge. The method of the study (qualitative or quantitative) was used to categorize the selected studies (qualitative, quantitative, or mixed methods). Two researchers judged the quality of studies using the Mixed Methods Appraisal Tool (MMAT; Version 2018).^29^ The GRADECER-Qual^30^ was used to assess confidence in synthesized qualitative findings.

#### 4.2.1 Data extraction

Research reports were constructed by extracting data from selected qualitative, quantitative, and mixed methods studies using criteria suggested by Sandelowski and Barrosso.^31^ Criteria extracted for the research reports included the study’s demographic features, research purpose and questions, sampling strategy and techniques, sample size and composition, data collection sources and techniques, data analysis and techniques, validity of study, findings, and protection of human subjects^31^ (p. 101). Extracting similar data for each research report facilitated the comparison of research findings and aided the researcher to assess the “nature” of how the included qualitative, quantitative, and mixed methods qualitative and quantitative study findings were related or similar based on the extracted criteria (research question, purpose, or findings) influences the method of synthesis (aggregation or configuration).^9^ Assessing the connections between the criteria assisted the researchers to examine whether qualitative, quantitative, and mixed methods’ study findings were complementary (linked observations and findings), confirming/refuting (address similar research questions and purposes with findings that repeat) or were in “direct opposition” across qualitative, quantitative, and mixed methods study findings^4^ (p. 6). Comparing qualitative and quantitative study findings, assisted researchers conclude that qualitative and quantitative findings were complimentary and could be synthesized within their “common domains” of research with “methods distinctive” to each domain and produce “separate synthesis products” (Sandelowski et al., 2006, p. 6). Therefore, separate synthesis of qualitative and quantitative study findings could be configured to produce a holistic understanding of unplanned readmission.

### 4.3 Step 3: Synthesis of qualitative study findings using meta-synthesis and applied thematic analysis

Sandelowski and Barroso’s^31^ meta-synthesis method using constant comparison and applied thematic analysis^28^was selected to synthesize the qualitative evidence. Applied thematic analysis is a methodological approach developed by Guest et al.^28^ comprised of “grounded theory, positivism, interpretivism and phenomenology” that assists researchers to develop explanations and understandings that can be used in practice settings (p. 15). To address the qualitative research question, the meta-synthesis offered a method to construct a “novel” integrated interpretation, explanation, and holistic understanding of qualitative research evidence^31^ (p. 151) from phenomenological, ethnographic, grounded theories, or descriptive study findings^31^ (p. 18) related to older persons with MCCs experiences of unplanned readmission. By synthesizing and integrating findings from diverse qualitative study approaches, findings from a meta-synthesis provided more categories of analysis and separate analysis of the categories. Additionally, meta-synthesis provided depth to the analysis so “penetrating interpretations” would describe and explain older people’s experiences of, understandings of, and factors influencing the event^31^ (p. 152; unplanned readmission). Using meta-synthesis assisted the researchers to generate a new explanation about what psychosocial processes occur during older persons with MCCs’ unplanned readmission experiences by linking aspects of the findings of many older peoples’ experiences through interpretative explanation.^31^

To understand the psychosocial processes that occurred during older persons with MCCs’ unplanned readmission experiences, two qualitative meta-syntheses methods were used. The two methods included “constant targeted comparison”^28^ ^,^ ^31^ and applied thematic analysis.^28^ Constant targeted comparison is a purposeful strategy that is used in meta-synthesis, ethnography, and applied thematic analysis to identify similarities and differences, illuminate attributes (defining and overlapping).^28^ ^,^ ^31^ We used constant targeted comparison to detect relationships between the constructed psychosocial processes of and factors (analytic themes) influencing older persons’ unplanned readmission experiences; congruent with the purposes of thematic analysis. Applied thematic analysis combines aspects of phenomenology and ethnography as evidenced in its aims to describe and “give voice to” the implicit and explicit themes from the data and identify how themes are related to each other^28^ (p. 12). Applied thematic analysis involved extracting and reducing data from the findings sections of included qualitative studies (research reports) and identifying descriptive and analytic themes related to the experiences^28^ of older persons unplanned readmission to hospital. The matrix was used by the researchers to first display and determine the similarity and areas of similarity between findings of the included qualitative studies (*n* = 5) and then to synthesize the qualitative evidence (descriptive and analytic themes).

#### 4.3.1 Descriptive themes

Text elements (processes) were analyzed and grouped for “similarity, dissimilarity, and relationships”^28^ (p. 50) and boundaries of segmentation or “key-word-in-context” (KWIC; p. 51). The “key-word-in-context” identified the context and focus of the theme in the text body and included the features of the theme that differentiated it from other themes.^28^ In the next step, codes or labels were developed, defined, and redefined. Codes or labels were described and linked theoretical and conceptual components, which helped describe and exemplify instances of a theme and in assisted to identify when the code should or should not be used^28^ (p. 54). To help sort the meaning of text into “categories, types and relationships of meaning” the research team developed a codebook^28^ (p. 52). The codebook was more than descriptive, as it assisted the researchers to analyze and explain what was common, different, and related in the instances, codes, and meaning of text.^28^

#### 4.3.2 Analytic themes

Structural and content coding was completed to contribute to the development of analytic themes by searching for words and reducing data.^28^ Structural coding identified the meaning (and description of the code label) and content coding was used to categorize text segments that were common, different, related, unclear, or identified speakers. Responses from individuals were compared across participant studies. Comparing responses and the content of codes helped researchers to determine how participants’ responses, descriptions, and explanations were similar or how events were related through causes, effects, or hierarchies^28^ (p. 64). This process was used to update or change decisions about the constructed codes. Analytic themes were generated from the descriptive themes using cues from the “meaning in text” to interpret and infer personal and social meaning from the dialogue^28^ (p. 14). This step was described as an “interpretive” mode of analysis and is epistemologically linked to phenomenology and grounded theory,^28^ similar to meta-ethnography being closely linked to grounded theory.^31^

### 4.4 Step 4: Synthesis of quantitative study findings using vote counting

In the synthesis of quantitative data, researchers used systematic review procedures to address the research question. The heterogeneity of included quantitative studies (*n* = 5) was assessed using Page et al.’s^32^ criteria related to research objectives, methodologies, study settings, and procedures of statistical analysis. Dissimilarity among the studies resulted in researchers analyzing quantitative data using vote counting to synthesize data about the factors influencing unplanned readmission. A matrix was used to display the quantitative data. A positive vote (1) was assigned to factors with significant positive results (*p* < 0.05), a negative vote (−1) was assigned to factors with significant negative results (*p* > 0.05), and a neutral vote (0) was assigned to results with no significant findings.^15^ Then, similar results were grouped into categories and summed by the researcher. Categories with the most votes were identified as having more strength^33^ (p. 359) and considered to be quantitative factors influencing unplanned readmission. Risk factors and factors associated with unplanned readmission included: age, living with someone less than 15 years old, number of hospital admissions or visits, primary discharge diagnosis, decreased ability to care for self, intensity of symptoms (depression, anxiousness, drowsiness, and shortness of breath), discharge home without home health care, and being asked about having needed help at home^15^ (pp. 5805–5806). After the research evidence from the qualitative and quantitative domains were separately synthesized, the synthesis findings were integrated.^4^

### 4.5 Step 5: Integration of synthesized qualitative and quantitative evidence

**Figure 2:**
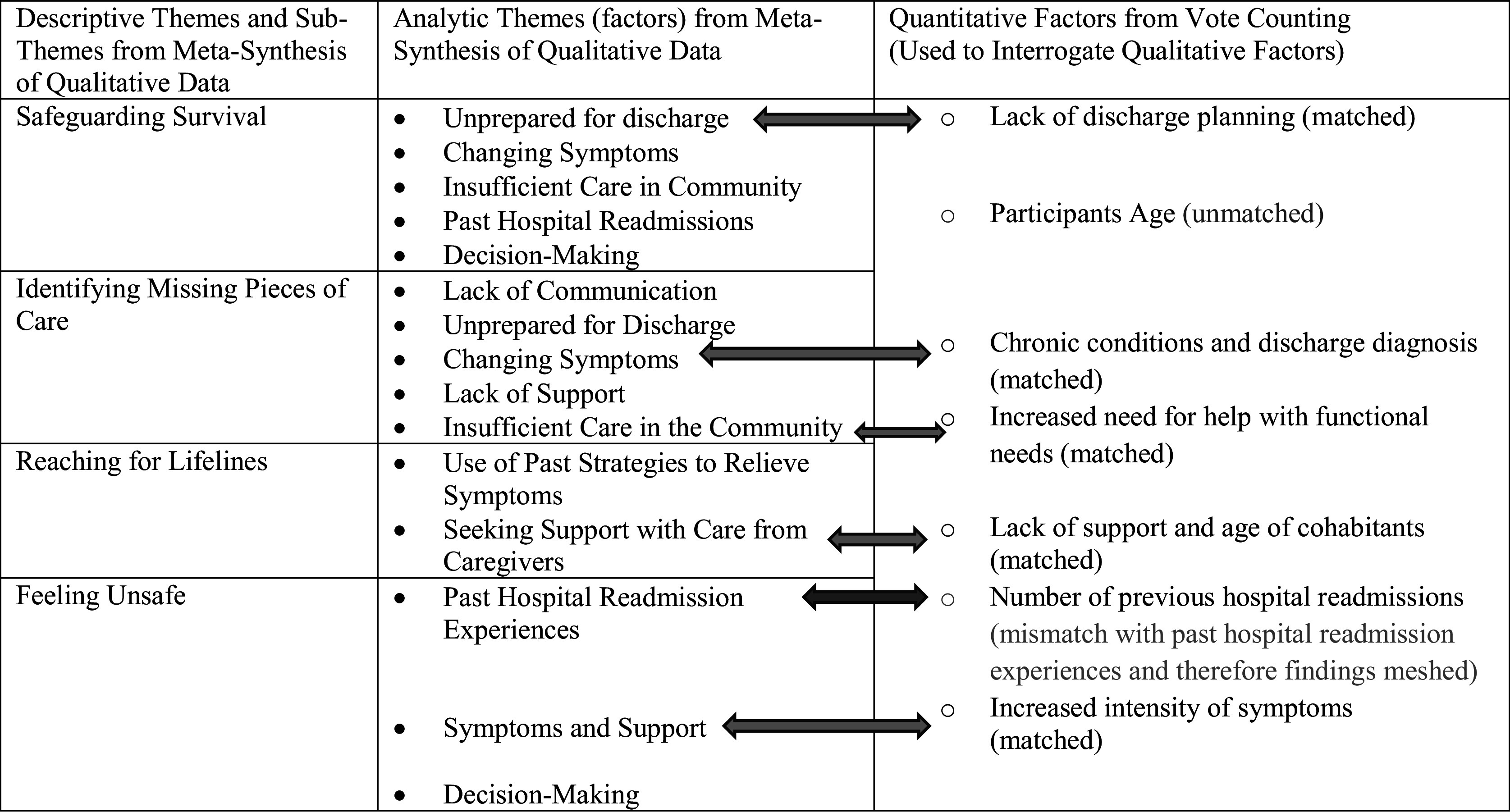
Process of using a matrix to identify matches between psychosocial processes qualitative and quantitative factors. Note: The processes of matching, mismatching, and gaps in themes from the qualitative meta-synthesis findings and quantitative meta-analysis findings are illustrated. The straight blue coloured arrows show the match between qualitative and quantitative factors and the green arrow shows the mismatch between qualitative and quantitative factors.

Two steps were completed to integrate qualitative meta-synthesis findings with quantitative synthesized (vote counting) findings to address the overarching question: What is the experience of unplanned readmission within 30 days of being discharged home for older persons with MCCs? In the Step 5a, the findings from Step 3 (the psychosocial processes and analytic themes) and findings from Step 4 (factors identified from vote counting) were placed into a matrix to assist with analyzing the evidence. The methodological and conceptual matrix assisted in evaluation of similarities (homogeneity) and differences (heterogeneity) between the qualitative evidence (psychosocial processes (descriptive themes) and qualitative factors influencing unplanned readmission across the qualitative studies (*n* = 5) and quantitative evidence (factors identified from the quantitative studies; *n* = 5). The matrix assisted the researcher to explore, illustrate, and identify which findings (qualitative and quantitative) were similar (matches) between the factors that influenced the experience of unplanned readmission and which factors (qualitative and quantitative) were different, specifically factors that did not influence unplanned experiences (mismatches), and where gaps existed in the findings. Mismatches in findings occur when individual findings are dissimilar, do not validate or confirm each other, and are not able to be combined.^9^ Thus, findings that were mismatched or dissimilar were “pieced-together” by the synthesizing called meshing (p. 9). Meshing was used because the findings that were placed together (age as a variable and factor influencing unplanned readmission) were placed together in the reviewed research reports^9^ (p. 9). Figure 2 is an example of the matrix that was used. The first column is the themes from the meta-synthesis of the qualitative data. The second column presents the themes from the qualitative findings that reflect the influences of the experience. The last column has the factors from the vote counting within the quantitative findings. The straight blue arrows show where the qualitative findings and quantitative finding match. The straight green arrow illustrates where there is a mismatch. For example, the qualitative factor lack of support and quantitative factor, age of cohabitants, was a match as both influenced unplanned readmission. Cohabitants less than 15 years old influenced older persons’ unplanned readmission, similar to lack of support. From the figure you will see the participants’ age was unmatched. Age was unmatched because we identified it as a factor influencing unplanned readmission and calculated participants’ age as a variable. Not all the qualitative factors match the quantitative, illustrating the benefit of using a mixed methods design to get a more complete representation of the experience. Age was identified as a factor in synthesized quantitative evidence as influencing unplanned readmission but was not identified in the QES. The reason for age not being identified in the QES may be because age was a variable used to describe the participants in the MMSR. On average, participants were 75.5 years old.^15^ Gaps identified between the qualitative themes and quantitative factors included: lack of communication between older persons and health care providers, older persons’ use of past strategies to relieve symptoms, and older persons’ decision making about seeking unplanned readmission to restore their safety.

Cross-study synthesis matrix assisted the researchers to determine how to juxtapose the findings^4^ ^,^ ^11^ by aggregation, configuration, and meshing results.^9^ For example, similar synthesized factors from vote counting were aggregated by vote counting and the meta-synthesis findings at the study level and with linking and meshing findings through configuration.^9^ Similar qualitative and quantitative synthesis findings that matched were aggregated as they addressed the same factors of unplanned readmission^9^ (p. 7). In the next Step 5b, the findings that were matched, not matched, or had gaps assisted in defining and describing the factors associated with unplanned readmission from older persons with MCCs’ experiences. Thus, the matrix illustrated the process of how findings were integrated.^15^

Linking or meshing are processes of the research synthesis of configuration.^9^ In configuration, sets of aggregated findings are linked or meshed^9^ (p. 9). Configuration assisted the researcher to understand the relationships between findings that “contradict, extend, explain, or modify” each other (p. 9). The process of linking or meshing occurred after the researcher reflected on matches, mismatches, and gaps.^5^ For example, older persons with MCCs’ previous hospital admission experiences were identified as a factor in the meta-synthesis findings and the number of unplanned readmissions was identified as a factor from the synthesized quantitative evidence. Although these factors appeared to be similar in their focus on unplanned readmission; the factors were different with the qualitative factor concentrated on the older persons’ experiences and the quantitative factor fixed on the number of times the event occurred.^15^As a result, the qualitative and quantitative evidence about unplanned readmission was not a mismatch but were a match and identified as the factor of previous hospital readmission experiences.^15^ From the qualitative data, unplanned readmission experience was a factor that influenced unplanned readmission but was a mismatch to previous hospital admission experiences. Therefore, meshing was used to merge findings that were “never placed together in researcher reports reviewed”^4^ (p. 9) and place these factors in the psychosocial process that influenced older persons’ feelings of being unsafe and unplanned readmission. Further, older persons previous hospital admission was linked to older persons’ experiences of unplanned readmission. The final overarching theme, themes and factors about older persons with MCCs’ experiences with unplanned readmission were identified^15^ and assisted with understanding the “big picture”^9^ (p. 10) of unplanned readmission experiences in older persons with MCCs. Specifically, how older persons’ feelings of safety are compromised by the increasing intensity and unmanageability of their symptoms and that intervention older persons use to maintain their safety and survival is unplanned readmission.^15^ The matrix also assisted researchers to identify factors that nurses could use to initiate discussions about and assess older persons and their family caregivers unplanned readmission experiences. The identified factors may be used as a checklist to develop discharge or nursing care plans, determine the home health care needs of older persons, or develop interventions to address older persons’ unmet psychosocial, physical, and safety needs, and unpleasant emotions as they transition from hospital to home.^15^

The process of configuration helped researchers understand how qualitative data (factors) influenced or explained “conditions for the occurrence of events” (themes) of unplanned readmission^4^ (p. 7). Coatsworth-Puspoky et al.^15^ found that as older persons experienced each psychosocial process, they felt more unsafe from the intensity and unmanageability of their symptoms. Attaining unplanned readmission was needed to relieve symptoms and distress, promote recovery, restore safety, and survive. Three psychosocial processes or conditions that older persons with MCCs’ experienced included: identifying missing pieces of care, reaching for lifelines, and feeling unsafe. Each of the psychosocial processes was influenced by integrated factors. For example, the factors that influenced older persons identifying missing pieces of care included burden of chronic conditions and discharge diagnoses, lack of discharge planning, and increased assistance with functional needs. Next, older persons reached for lifelines, but found a lack of support. Finally, older persons experienced feeling unsafe which was influenced by an increase in the intensity of symptoms and previous hospital readmission experiences (p. 5809).

### 4.6 Optimization of findings

Optimization of findings occurred by using descriptive, interpretive, theoretical, pragmatic, and consensual validity to ensure the validity of findings.^31^ Findings were optimized with an audit trail, regularly scheduled meetings with team members (to ensure consensual validity),^28^ ^,^ ^31^ drawing on the expertise of peers for review of findings,^31^ and using a reflexive journal.^28^ Weekly meetings (by phone, zoom, email, or correspondence), interpretation of the findings at each stage, and how the findings were integrated in the final stage were documented by the first author using an audit trail.^28^ ^,^ ^31^ Understanding about the model of unplanned readmission was maximized by reflecting on the audit trail and the process associated with integrating older persons’ unplanned readmission (quant) into the QUAL findings of the unplanned readmission processes. Consistent with applied thematic analysis, the audit trail provided the researcher with a document that reflected the changes that occurred during the research process, such as changes in the codebook, rationales, and how the systematic method was maintained, in keeping with applied thematic analysis^28^ (p. 94).

The codebook was used to “extend, revise, and test ideas” within the evidence of data; and was updated as definitions changed^28^ (p. 75). During the third synthesis, negotiated consensual validity, or the collaborative social processes (agreeing, disagreeing, reasoning, explaining, and negotiating)^31^ were used by the team. During the weekly team meetings, this type of validity occurred between the research team members. Similarly, emails assisted the first author to be clear about the explanations and judgments surrounding how research findings were integrated. Consensual validity was documented in the audit trail to reflect how consensus was met or not met by team members and also in the code book and reflexive journal.^28^ The matrix was a joint display tool that we used to visualize the findings, share the visualization about how findings were integrated and interpretated within the team, and disseminate the evidence in our publication. This assisted to ensure transparency in the analysis of data and findings.^34^

In the absence of an exemplar MMSR that prioritized QES, we documented our method and illustrated our framework. We used the key principles articulated by Harden and Thomas^5^: transparency (describing the approach and method used to integrate and synthesize study findings); error avoidance (the strategies used in reviewing to uphold rigor); matching and adapting review methods (the process of matching method of synthesis and integration to included studies); and identifying our perspectives and intent about research and the purpose of the studies^5^ (p. 12). We followed the philosophical foundations of pragmatism and viewed the qualitative and quantitative research studies as complimentary, but “a-paradigmatic” in that the studies (qualitative and quantitative) could not be analyzed together and were therefore independently and separately synthesized^5^ (p. 28). However, the strength of the findings from including both qualitative and quantitative studies was complimentary because the two types of studies provided a dialectal perspective in bringing different perspectives to answer the research questions and in the final stage of analysis, use the different perspectives for the purposes of interrogation.^5^ Articulating the procedures followed to complete this study is important for the development of methodological knowledge and consistency in research methods.

## 5 Discussion

A detailed description, illustration, and explanation of the methodological procedure developed to prioritize QES findings in a MMSR study are presented and illustrated. The MMSR design we utilized illustrates the “power” of combining the synthesized qualitative and quantitative evidence using integration to understand the complex phenomenon^3^ of older persons with MCCs’ unplanned readmission experiences. Combining stories and numbers from the unplanned readmission experiences of older persons with MCCs assisted researchers to develop new understandings about and insights into the psychosocial processes and factors that influence the unplanned readmission event.^4^ Therefore, qualitative data was not viewed “as an adjunct” or having “an accessory role” to quantitative data^4^ (p. 5), but as having utility in explaining the psychosocial process and factors that older persons identify as influencing their unplanned readmission, a “facet of human experience unreachable with quantitative methods” and an method that can assist with developing patient-centered interventions, increasing practical significance of quantitative research findings^4^ (p. 5).

In our MMSR procedure, screening, collection, appraisal, and analysis of qualitative and quantitative evidence were completed systematically and independently with synthesis of qualitative evidence prioritized or completed before the quantitative evidence synthesis. Prioritizing QES in an MMSR advances and expands the use and the potential power of qualitative evidence^17^ ^,^ ^26^ ^,^ ^35^ and our understanding of older persons’ experiences to develop nursing knowledge and influence nursing practices. Our study was unique as the QES was prioritized and synthesized using meta-synthesis^31^ and applied thematic analysis.^28^ Both meta-synthesis and applied thematic analysis are methods grounded in phenomenology, ethnography, and grounded theory.^28^ ^,^ ^31^ Philosophically, the overarching paradigm was pragmatism. The paradigm of qualitative evidence (constructivism) was prioritized and quantitative evidence (positivism) was used to interrogate the qualitative evidence in the final stage of analysis. The philosophical stance taken by researchers and research question used to prioritize evidence synthesis raises important considerations for researchers using the MMSR design.

To address the aim of the study, we developed three research questions: one overarching question and two questions to guide analysis. The overarching question was developed to understand and construct knowledge about “What is known about the experience of unplanned readmission within 30 days of being discharged home among older persons with MCC?” The two questions: what are the psychosocial processes that occur during older persons with MCCs unplanned readmission experiences and what are the factors associated with older persons with MCCs unplanned readmission were developed to construct knowledge about different aspects of older persons with MCCs’ experiences of unplanned readmission. Consistent with Harden and Thomas’^5^ MMSR method, each question used different types of studies and was independently synthesized using different synthesis methods. For example, to understand the psychosocial processes of the unplanned readmission experiences we synthesized qualitative data from qualitative studies (no mixed methods studies were identified to be included) synthesized the data using meta-synthesis and applied thematic analysis. To identify the factors influencing older persons with MCCs unplanned readmission experiences, data from quantitative studies (no mixed methods studies were identified) were synthesized using vote counting. To address the overarching question, synthesized qualitative and quantitative evidence were synthesized using a matrix. Three syntheses methods were used to answer three research questions related to older persons with MCCs’ unplanned readmission experiences. Harden and Thomas^5^ suggested that their MMSR could be expanded by varying how synthesis methods are weighted, increasing number of independent syntheses, and altering how independent syntheses are sequenced. Variations in the MMSR designs reinforce the need for an overarching research question to guide the development of research questions about the experience that is being examined by researchers. Illustrating the “review process” undertaken by researchers using a diagram will be critical for understanding, evaluating, and advancing the knowledge in MMSR methods.

In the presented MMSR, QES was prioritized and used meta-synthesis^31^ with applied thematic analysis^28^ methods. By prioritizing the QES, the feelings, descriptions, and quotes (voices) associated with the experiences^1^ ^,^ ^18^ of older persons with MCCs (*n* = 12, 336) across five studies from four countries were combined to explain and understand the complex phenomenon of unplanned readmission “in terms of the meanings people bring to them”^2^ (p. 3). Synthesizing evidence using a meta-synthesis method^31^ allowed the researchers to synthesize and compare included study findings individually and collectively to extend nursing knowledge about unplanned readmission “beyond the findings in primary studies”^26^ (p. 320) with the intent of “resolving patient and practice problems”^24^ (p. 370). In our study, the voices (quotes) and experiential knowledge of older persons with MCCs^15^ were used to construct and explain the psychosocial processes that older persons with MCCs experienced during unplanned readmission and identify qualitative factors that influenced unplanned readmission. Prioritizing the older patients’ health care experiences during unplanned readmission using a meta-synthesis method and applied thematic analysis assisted researchers to use older persons’ health care experiences about unplanned readmission to develop an in-depth understanding about the complex phenomenon: psychosocial processes and factors influencing unplanned readmission. The results presented a new understanding and illustration about how older persons actively prioritize their psychological, physical and emotional safety, recovery, and health care needs by using unplanned readmission as an intervention to safeguard their survival.^15^ The findings were positioned to explain^4^ (p. 7) factors and conditions within each stage of the psychosocial processes.

The psychosocial processes of and factors influencing unplanned readmission included: identifying missing pieces of care (burden of chronic conditions, discharge diagnosis, lack of discharge planning, increased assistance with functional needs), reaching for lifelines (lack of support), and feeling unsafe (increase in intensity of symptoms, previous hospital readmission experiences).^15^ Meta-synthesis findings and factors related to unplanned readmission may be useful for health care providers to develop care practices that are “personalized” and “tailored”^36^ (p. 909) to the older persons’ psychological and emotional safety health care needs across the care continuum.^37^ In the second synthesis, factors that influenced unplanned readmission were identified from synthesizing quantitative evidence (*n* = 5) using vote counting. The identified quantitative factors were compared to and explained using the qualitative factors that influenced unplanned readmission and were then integrated into the psychosocial processes. Thus, older persons’ perspectives and understandings were synthesized and integrated into the findings from the MMSR study which was consistent with our study purpose and research questions.

Our study purpose focused on a qualitative overarching research question to explain and understand the patient experience of unplanned readmission (the psychosocial processes and factors of), in contrast to using qualitative data to explain the effectiveness or implications of interventions.^5^ Although it is important to understand the effectiveness of health care interventions and clinical decisions,^14^ it is also important to understand older persons’ experiences with the health care system, such as unplanned readmission, and what unmet needs older persons have and how the event of unplanned readmission influence older persons’ health and well-being. The knowledge developed from our study identified that the psychosocial processes experienced during unplanned readmission increased in intensity and symptoms increased in unmanageability, jeopardizing older persons’ feelings of safety at home.^15^

Describing the methodological steps raises questions about the MMSR philosophical underpinnings and questions about the influence of the study purpose on when and how qualitative evidence is synthesized in MMSR research designs that prioritize QES. Tricco et al.^38^ challenged researchers to utilize different synthesis methods to address one research question and how findings across syntheses methods vary. The researchers hypothesized that this knowledge would assist researchers to align research questions with knowledge synthesis methods. Building on Tricco et al.’s^38^ challenge, they also argued for more research about MMSR methods that prioritize QES to determine how research questions influence the development of findings and align with paradigmatic stances of qualitative and quantitative research. Similarly, additional research is needed to understand the range of synthesis methods and their influence on the knowledge that is constructed within MMSR methods.

Harden and Thomas^5^ used thematic synthesis^39^ to synthesize qualitative data. The three-stage method resulted in descriptive and analytic themes. The construction of descriptive and analytic themes was identified as a “crucial step” as the synthesized data was needed to complete the final (Step 5) synthesis^5^ (p. 19). Similarly, we also synthesized qualitative data resulting in themes and factors which would be used in Step 5. Thus, to address the research question in Step 3 (What are the psychosocial processes that occur during older persons with MCCs’ unplanned readmission experiences?), we selected the meta-synthesis method instead of the meta-summary. The meta-summary, in contrast to the meta-synthesis, was described as a superficial report or summary of the themes and did not result in “penetrating interpretations” which were needed to describe and explain older people’s experiences of, understandings of, and factors influencing the event^31^ (p. 152) (unplanned readmission). We found that the meta-synthesis method supported us to construct a new holistic interpretation and conceptual in-depth description of the experience of unplanned readmission by linking aspects of the findings of many older peoples’ experiences through interpretative explanation.^31^ Using meta-synthesis and applied thematic analysis assisted the researchers to develop a figure or model^26^ to illustrate the progression of the psychosocial processes and the factors that influenced each stage of the process between hospital admission and unplanned readmission^15^ adding to our understanding of unplanned readmission.

Prioritizing the QES in MMSR was significant as it strengthened the voices^24^ ^,^ ^28^ of older persons with MCCs and produced a model that was constructed from older persons’ experiences about unplanned readmission. Prioritizing QES also identified differences in language between older persons and health care providers and about factors that influence unplanned readmission^15^ as predicted by Sandelowski et al.^24^ These findings may be useful for health care providers when initiating discussions with older persons about discharge, returning home, their unplanned readmission experiences, or when listening to older persons experiences and health care needs.

Agreement about language is needed amongst researchers related to MMSR studies. Researchers need to use consistent language not only in naming and defining of mixed method research reviews^2^ ^,^ ^16^ and QES studies but also about knowledge about reporting MMSR studies,^14^ and the evidence-based medicine movement.^2^

These findings may contribute to establishing “best practices to guide future reviews” by providing clarity about the procedure and decisions that were used gather, analyze, and compile the results throughout this method^16^ (p. 556), a current challenge in the literature related to synthesis methods.^38^ The title of the completed study is identified as a MMSR and the reason for using this review design was articulated by the researchers. Combined, these two factors enable other researchers to identify it within the published literature and evaluate the “value of”^16^ (p. 547) MMSR to the development of knowledge in nursing.

## 6 Conclusion

This paper presents a new MMSR method to prioritize QES (meta-synthesis findings) in a MMSR. This method presents a review method or “dialectic stance” of using the findings of one synthesis (quantitative) to interrogate the second synthesis (qualitative)^5^ (p. 29). Prioritizing the synthesis of qualitative evidence with a method suitable to analyze older persons experiences and perspectives advances the utilization and development of QES^17^ in MMSR studies. Prioritizing QES in the MMSR assists researchers to understand complex clinical problems from a unique perspective-the people who experience these problems. It provides researchers with the opportunity to construct a holistic understanding of the psychosocial processes and factors influencing unplanned readmission using the language, and subjective experiences^28^ of older persons with MCCs.^15^ This MMSR design to synthesize and integrate data expands our current understanding of MMSR methods and methodologies and resulted in a unique understanding the psychosocial processes and factors older persons experienced during unplanned readmission.

## Data Availability

Data sharing not applicable to this article as no datasets were generated or analyzed during the current study.
